# LncRRIsearch: A Web Server for lncRNA-RNA Interaction Prediction Integrated With Tissue-Specific Expression and Subcellular Localization Data

**DOI:** 10.3389/fgene.2019.00462

**Published:** 2019-05-28

**Authors:** Tsukasa Fukunaga, Junichi Iwakiri, Yukiteru Ono, Michiaki Hamada

**Affiliations:** ^1^Department of Electrical Engineering and Bioscience, Faculty of Science and Engineering, Waseda University, Tokyo, Japan; ^2^Department of Computer Science, Graduate School of Information Science and Technology, The University of Tokyo, Tokyo, Japan; ^3^Graduate School of Frontier Sciences, University of Tokyo, Chiba, Japan; ^4^Artificial Intelligence Research Center, National Institute of Advanced Industrial Science and Technology, Tokyo, Japan; ^5^IMSBIO Co., Ltd., Tokyo, Japan; ^6^Computational Bio Big-Data Open Innovation Laboratory, National Institute of Advanced Industrial Science and Technology, Tokyo, Japan; ^7^Institute for Medical-oriented Structural Biology, Waseda University, Tokyo, Japan; ^8^Graduate School of Medicine, Nippon Medical School, Tokyo, Japan; ^9^Center for Data Science, Waseda University, Tokyo, Japan

**Keywords:** lncRNA, RNA-RNA interaction, web server, tissue-specific expression, subcellular localization

## Abstract

Long non-coding RNAs (lncRNAs) play critical roles in various biological processes, but the function of the majority of lncRNAs is still unclear. One approach for estimating a function of a lncRNA is the identification of its interaction target because functions of lncRNAs are expressed through interaction with other biomolecules in quite a few cases. In this paper, we developed “LncRRIsearch,” which is a web server for comprehensive prediction of human and mouse lncRNA-lncRNA and lncRNA-mRNA interaction. The prediction was conducted using RIblast, which is a fast and accurate RNA-RNA interaction prediction tool. Users can investigate interaction target RNAs of a particular lncRNA through a web interface. In addition, we integrated tissue-specific expression and subcellular localization data for the lncRNAs with the web server. These data enable users to examine tissue-specific or subcellular localized lncRNA interactions. LncRRIsearch is publicly accessible at http://rtools.cbrc.jp/LncRRIsearch/.

## 1. Introduction

Long non-coding RNAs (lncRNAs) were initially considered to be transcriptional noise or experimental artifacts, but recent research has revealed that lncRNAs play important roles in various biological processes, such as cell differentiation (Fatica and Bozzoni, 2014) and functioning of the immune system (Carpenter et al., 2013). While large-scale RNA sequencing studies have discovered several tens of thousands of lncRNAs in the human transcriptome (Iyer et al., 2015; Hon et al., 2017), the function is known in detail for only a small number of lncRNAs (Quek et al., 2014; de Hoon et al., 2015). To understand the molecular mechanisms of complex biological systems, elucidating the functions of more lncRNAs is an important research topic.

Recent discoveries of lncRNA-mRNA interactions regulating biological processes (Gong and Maquat, 2011; Kretz et al., 2013; Abdelmohsen et al., 2014) suggest that comprehensive lncRNA-mRNA interaction predictions are helpful for the estimation of lncRNA function. Several databases or web services have been developed for the function prediction based on lncRNA-mRNA interactions, but there are no web services for comprehensive prediction of human and mouse lncRNA interaction. RAID contains some lncRNA-mRNA interaction data taken from the literature, but the number of interactions is limited and comprehensiveness is low (Yi et al., 2017). RISE includes experimentally validated lncRNA-RNA interactions based on high-throughput sequencing methods (Lu et al., 2016; Nguyen et al., 2016), but the number of lncRNA interactions is also limited (Gong et al., 2017). The database compiled by Terai et al. (2016) contains predicted lncRNA-mRNA and lncRNA-lncRNA interaction data at transcriptome scale, but the database does not store more than one local base-pairing interaction for each lncRNA-RNA interaction. In addition, the database includes only human lncRNA-RNA interactions.

To address these shortcomings, we have constructed the LncRRIsearch, which is a web server for comprehensive prediction of human and mouse lncRNA-mRNA and lncRNA-lncRNA interactions. We applied RIblast to human and mouse transcriptome to predict RNA-RNA interactions (Fukunaga and Hamada, 2017). LncRRIsearch provides multiple local base-pairing interactions predicted by RIblast for each lncRNA-RNA interaction. In addition, unlike previous databases or web services, we integrated tissue-specific RNA expression and subcellular localization data of lncRNAs with our web service. These data help us to verify the correctness of the predicted interactions. Actually, we showed the tissue-specificity information improves the prediction accuracy for lncRNA-RNA interactions in previous research (Iwakiri et al., 2017). LncRRIsearch is freely accessible at http://rtools.cbrc.jp/LncRRIsearch/.

## 2. Materials and Methods

### 2.1. Dataset of lncRNA and mRNA Sequences

We downloaded human and mouse RNA sequences from GENCODE version 25 and M14, respectively (Harrow et al., 2012). While we used all lncRNA transcript sequences in our analysis, we used the longest mRNA transcript for each gene to reduce the size of the dataset. In addition, we excluded transcripts in the pseudoautosomal region on the Y-chromosome from the analysis. As a result, we obtained 27,674 lncRNA and 20,360 mRNA transcripts as human RNA dataset, and 16,113 lncRNA and 22,468 mRNA transcripts as mouse RNA dataset. Note that LncRRIsearch contains an additional 175 mRNA and 3,776 lncRNA transcripts in comparison with the database previously compiled by Terai et al. (2016) as human RNA dataset. This difference is derived from the version update of GENCODE.

### 2.2. Prediction of lncRNA-RNA Interactions

RNA-RNA interaction prediction for long RNAs is time-consuming calculation, and even the fastest programs at present cannot be predict the interactions in real-time. Therefore, we predicted comprehensive human and mouse lncRNA-mRNA and lncRNA–lncRNA interactome in advance, and stored the interaction results in MySQL database. By selecting a query RNA or a target RNA, users can obtain pre-calculated prediction results of the selected RNA.

We used the RIblast program, which has been recently developed by our group, for comprehensive RNA-RNA interaction prediction (Fukunaga and Hamada, 2017). RIblast predicts local base-pairing interactions based on interaction energy that is computed by using both accessibility energy and hybridization energy. Briefly, RIblast considers both effects on stabilization energy derived from hybridization between two RNA sequences and the energy for preventing the formation of intramolecular double-stranded structure. (If an RNA region forms double-stranded structure in the secondary structure, the region does not tend to interact with the other RNA molecules via base-pairing.) RIblast output multiple candidates for local base-pairing interactions for each RNA-RNA pair. The threshold interaction energy was set to −12 or −16 kcal/mol. We regarded the query and target RNA pairs (A, B) and (B, A) as being different because RIblast predicts slightly different interactions for these pairs. Users can sort target transcripts for each query transcript by two criteria: MINENERGY and SUMENERGY. MINENERGY denotes the minimum interaction energy of local base-pairing interaction among all interactions between the query RNA and the target RNA. SUMENERGY means the sum of all interaction energies of local base-parings for the RNA-RNA pair.

We investigated whether the experimentally validated lncRNA-mRNA interactions were predicted by RIblast. We verified that RIblast predicted human 1/2-sbs RNA (ENST00000548810) and SERPINE1 (ENST00000223095) interaction, and human 1/2-sbs RNA and ANKRD57 (ENST00000356454) interaction (Gong and Maquat, 2011). In addition, human 7SL RNA (ENST00000635274) and TP53 mRNA (ENST00000617185) interaction (Abdelmohsen et al., 2014) was also predicted by RIblast. As we did not predict mRNA-mRNA interactions, LncRRIsearch does not provide human TINCR-mRNA interactions (Kretz et al., 2013) (TINCR ENST00000448587) was annotated as mRNA in GENCODE ver.25). In summary, our prediction results include experimentally validated lncRNA-mRNA interactions for lncRNAs.

### 2.3. Expression Analysis for Tissue-Specific lncRNA-RNA Interaction

Expression levels of human lncRNA and mRNA genes were estimated from RNA-seq data derived from five international consortia. The first RNA-seq dataset was derived from 32 tissues collected from 122 human individuals, which was produced by the Human Protein Atlas Project (Expression Atlas ID: E-MTAB-2836) (Uhlén et al., 2015). The second RNA-seq dataset was derived from 30 representative tissues, released by the GTEx Consortium (Expression Atlas ID: E-MTAB-2919) (GTEx Consortium, 2015). The third RNA-seq dataset was produced by the Human Body Map Project from 16 tissues (Expression Atlas ID: E-MTAB-513) (Cabili et al., 2011). The fourth RNA-seq dataset, derived from 19 tissues isolated from fetuses with congenital defects, was released by the Epigenome Roadmap Project (Expression Atlas ID: E-MTAB-3871) (Kundaje et al., 2015). The last RNA-seq dataset, the largest collection of primary cells, was derived from 56 tissues produced by FANTOM5 project (Expression Atlas ID: E-MTAB-3358) (Forrest et al., 2014). Note that the second RNA-seq dataset originally contained 53 tissues derived from several cell lines and subregions of a single tissue. To reduce the number of redundant cell types, 30 representative tissues were arbitrarily selected.

In addition, expression levels of mouse lncRNA and mRNA genes were also estimated from RNA-seq data. The first RNA-seq dataset was derived from nine tissues harvested from an adult male C57BL/6 mouse (Expression Atlas ID: E-GEOD-74747) (Huntley et al., 2016). The second RNA-seq dataset was derived from three mouse strains (C57BL/6, DBA/2J, and CD1) (Expression Atlas ID: E-MTAB-2801) (Merkin et al., 2012). In this dataset, gene expression data across eight (C57BL/6 strain) or nine mouse tissues (DBA/2J and CD1 strains) is available.

Tissue-specificities of lncRNA and mRNA genes were investigated based on an outlier analysis of the RNA-seq data using ROKU (Kadota et al., 2006). For each lncRNA and mRNA gene, the tissues in which the gene was specifically expressed were detected based on its extremely high or low expression levels in one or a few tissues. These tissue-specificity data allow the user to investigate the tissue-specific lncRNAs which regulate the expression levels of their target mRNAs through the base-pairing interactions. The tissue-specific lncRNA-RNA interactions derived from the aforementioned five human RNA-seq datasets and four mouse RNA-seq dataset are provided in LncRRIsearch (Tables S1–S9).

### 2.4. Integration With Subcellular Localization Data to LncRRIsearch

Subcellular localization dataset was downloaded from the LncAtlas database (Mas-Ponte et al., 2017). This dataset includes 15 human cell-line subcellular localization data, and the localization was quantified by “relative concentration index” (RCI), which was defined as *log*_2_-transformed ratio of FPKM between two expression data. For example, high cytoplasmic/nuclear RCI means that the transcript tends to localize in cytoplasm rather than nucleus. For 14 cell-lines, two types of RCIs (cytoplasmic/nuclear and nuclear/cytoplasmic RCIs) are included in the dataset. On the other hand, for the K562 cell-line, five types of RCI data (Chromatin/Nucleus, Nucleolus/Nucleus, Nucleoplasm/Nucleus, Cell membrane/Cytoplasm, and Insoluble fraction/Cytoplasm RCIs) are additionally included in the dataset. These subcellular localized RNA-RNA interactions are also provided in LncRRIsearch (Tables S10–S12). The detail of the dataset was described in the original publication (Mas-Ponte et al., 2017). Note that mouse subcellular localization data are not included in LncRRIsearch.

### 2.5. Database Organization

In LncRRIsearch, tissue-specific expression data and subcellular localization data were stored in a series of MySQL databases. For RNA-RNA interaction data, all pre-calculated SUMENERGY and MINENERGR scores were also stored in the databases, but the local base-pair data were not stored in the databases because the data size is too large. In the web service, the base-pairs are re-predicted by RIblast in real time when both the query and target RNAs are selected based on SUMENERGY or MINENERGY scores. However, because RIblast cannot predict interactions of long RNAs in real-time, base-pair prediction results for RNA sequences longer than 5,000 nt were stored in the databases, and the data is referenced in the web service.

## 3. Results

**Figure 1:**
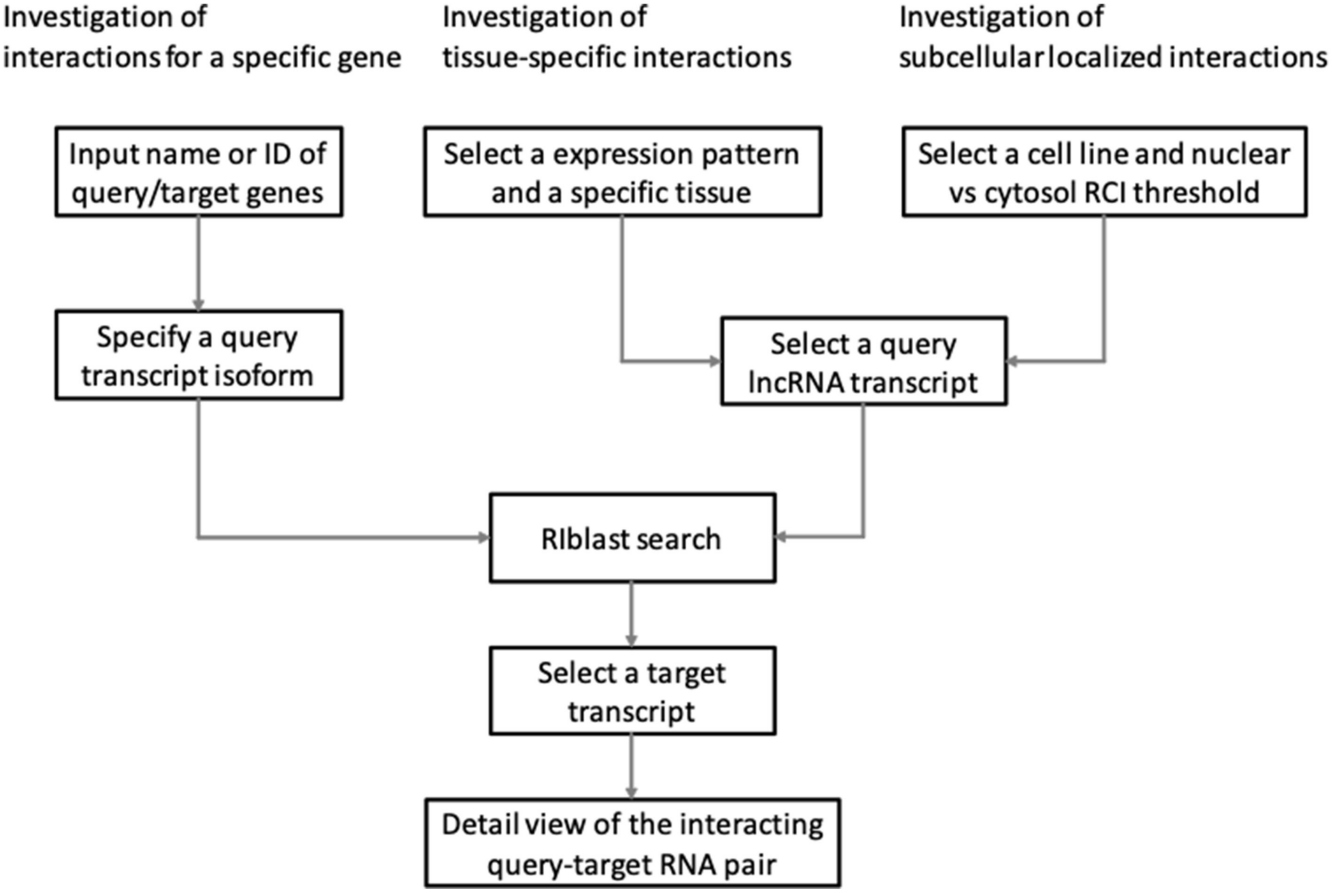
Three workflows for investigating lncRNA–RNA interaction using LncRRIsearch.

LncRRIsearch provides three types of interaction prediction method (Figure 1): a name/ID based method, an expression pattern-based method, and a localization-based method.

### 3.1. Investigation of an RNA-RNA Interaction Based on Name or ID

**Figure 2:**
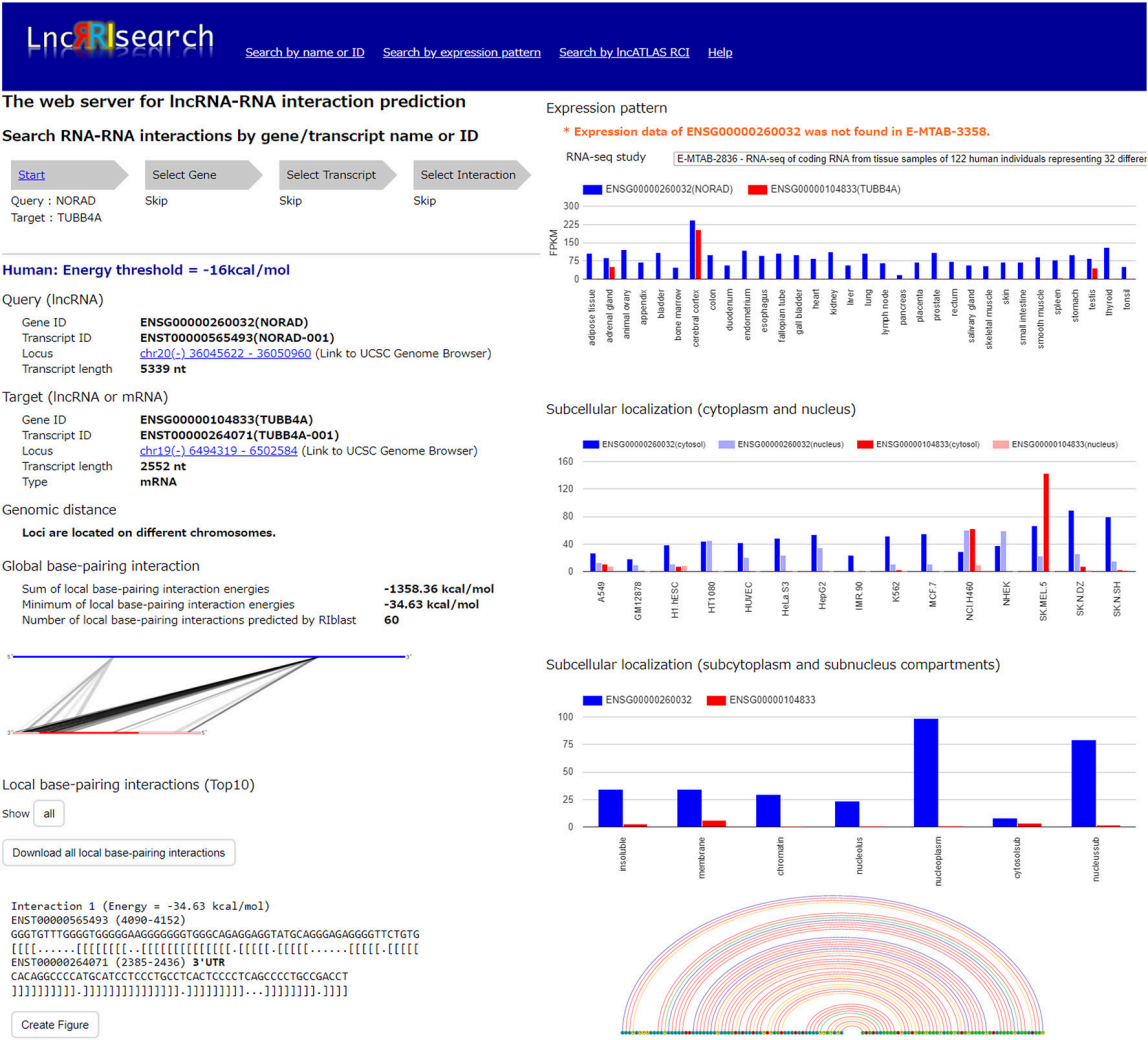
Investigation of lncRNA–RNA interactions using LncRRIsearch. In this example, detailed information about the interaction between NORAD lncRNA (used as query, Gene ID:ENSG00000260032, Transcript ID: ENST00000565493) and TUBB4A mRNA (used as target, Gene ID:ENSG00000104833, Transcript ID:ENST00000264071) is shown.

Users firstly select target species (human or mouse) and the energy threshold (−12 or −16 kcal/mol), and then inputs name or ID of genes or transcripts (Figure 1). LncRRIsearch supports GENCODE gene/transcript names or IDs as input type, and either query lncRNA or target lncRNA/mRNA is required as input RNA. After specifying a gene of interest, several transcript isoforms derived from the gene are listed for selection of a single lncRNA transcript if multiple isoforms are encoded in the gene. For the selected lncRNA transcript (query transcript), all interacting RNAs (target transcripts) predicted by RIblast are provided. After selecting a single target transcript, the details of the RNA-RNA interaction between query and target transcripts are described (Figure 2). In this step, all local base-pairing interactions are listed, and users can download the prediction results as a text file. In addition, the global base-pairing interaction is described as an image (The center left of Figure 2). In this figure, the query RNA and the target RNA are represented as a blue line and a red line, respectively, and the predicted interactions are displayed as gray or black lines between two RNAs. The color consistency means strength of interactions. For each local base-pairing interaction, text (output of RIblast) and a graphical view based on VARNA (Darty et al., 2009) are also provided (The lower left and the lower right of Figure 2).

### 3.2. Investigation of Tissue-Specific RNA-RNA Interactions

LncRRIsearch helps users to investigate lncRNA-RNA interactions exhibiting tissue-specific expression patterns (Figure 1). Users can select an RNA-seq dataset from four different RNA-seq studies and select a tissue of interest. For the selected tissue, one of three possible tissue-specific expression patterns for the query and target RNA transcripts should be selected: Query and target RNAs are specifically up-regulated in the same tissue; query RNAs are specifically up-regulated and target RNAs are down-regulated in the same tissue; or query RNAs are specifically down-regulated and target RNAs are up-regulated in the same tissue.

After selecting the tissue-specific expression pattern, the corresponding query and target RNAs predicted by RIblast are listed. In this step, once a query RNA is selected, the list of possible target RNAs is automatically updated for the selected query. By selecting the tissue-specific query and target RNAs, detailed information about interactions between the query and target RNAs is provided (Figure 2). In addition, the expression values of query and target RNAs are provided as a graphical view in the results page (The upper right of Figure 2).

### 3.3. Investigation of Subcellular Localized RNA-RNA Interactions

Users can investigate subcellular-localized human lncRNA-RNA interactions (Figure 1). Users firstly select a energy threshold and select a cell line of interest. For the selected cell line, a type of RCI and the threshold of RCI should be selected. Except for K562 cell line, users can choose which one of the nucleus/cytosol or cytosol/nucleus RCI. For K562 cell line, users have five choices of sub-compartments RCIs in addition to the above-mentioned two RCIs. The subsequent steps are the same as the investigation of tissue-specific RNA-RNA interactions. The RCI values of query and target RNAs are displayed as a graphical view in the results page (The center right of Figure 2).

## 4. Discussion

We developed LncRRIsearch, which is a web server for comprehensive prediction of human and mouse lncRNA-mRNA and lncRNA-lncRNA interactions including tissue-specific expression and subcellular localization data. There are two advantages of LncRRIsearch over other lncRNA-RNA interaction databases or web services; the comprehensiveness of interaction prediction and the ability to investigate tissue-specific or subcellular localized interaction patterns.

We envision three future improvements of LncRRIsearch. The first is the development of real-time RNA-RNA interaction prediction software. Although LncRRIsearch provides comprehensive human and mouse lncRNA-RNA interaction based on GENCODE version 25 and M14, novel lncRNAs will be discovered in the future. Real-time prediction would be useful for the discoverers of new lncRNAs to investigate their interactions. The acceleration of RNA-RNA interaction prediction is still an important research topic. One possible direction is the simplification of the energy model. RIblast uses a complete nearest-neighbor energy model in the search step, but some researchers have reported that the use of an approximated energy model produces a marked increase in the calculation speed in exchange for only a slight decrease in the prediction accuracy (Tafer et al., 2011; Wenzel et al., 2012; Alkan et al., 2017).

The second improvement is the integration of the results of RNA-RNA interaction detection experiments. Recently, several high-throughput sequencing methods for the exhaustive identification of RNA-RNA interaction sites have been developed, including PARIS (Lu et al., 2016) and MARIO (Nguyen et al., 2016). Although only a few lncRNA-related interactions have been detected in these experiments, simultaneously displaying predicted and experimentally verified interactions (where available) should be useful for users. In addition, such data will encourage researchers to develop machine-learning-based RNA-RNA interaction prediction programs.

The third improvement is an increase in the number of target species. This improvement would enable us to not only investigate the lncRNA interactions of newly added species but also compare lncRNA interactomes between species. Nguyen et al. recently showed that the conservation of experimentally confirmed lncRNA-RNA interaction regions is high, although lncRNA generally lacks sequence conservation (Nguyen et al., 2016). This means that conservation information should be useful for the verification of predicted lncRNA-RNA interactions.

## Data Availability

All datasets analyzed for this study are included in the manuscript and the Supplementary Files. LncRRIsearch is publicly available from http://rtools.cbrc.jp/LncRRIsearch/.

## Author Contributions

TF, JI, and MH conceived the study and wrote the manuscript. TF, JI, and YO processed the data. YO constructed the database. TF and JI equally contributed to this work. MH supervised this study. All authors read and approved the final manuscript.

### Conflict of Interest Statement

YO was employed by company IMSBIO CO., LTD. The remaining authors declare that the research was conducted in the absence of any commercial or financial relationships that could be construed as a potential conflict of interest.
