# Supplementary material for: LncRRIsearch: A Web Server for lncRNA-RNA Interaction Prediction Integrated With Tissue-Specific Expression and Subcellular Localization Data
Source: Front Genet. 2019 May 28;10:462. doi: 10.3389/fgene.2019.00462 (PMC6546843; doi:10.3389/fgene.2019.00462)
Supplement: Supplementary file 1 [file Data_Sheet_1.PDF]

Supplementary Material

Suplementary Tables

Table S1: Tissue-specific human lncRNA–RNA interactions in LncRRIsearch. LncRNA and protein-coding genes with expression levels $\geq$ 1 FPKM in at least one tissue were used for the prediction. Expression levels were derived from RNA-seq data of the Human Protein Atlas project (Expression Atlas ID: E-MTAB-2836). Tissue-specific expression of lncRNA and protein-coding genes was detected by ROKU. UP–UP: Query and target RNAs are specifically up-regulated in the same tissue. UP–DOWN: Query RNAs are specifically up-regulated and target RNAs are down-regulated in the same tissue. DOWN–UP: Query RNAs are specifically down-regulated and target RNAs are up-regulated in the same tissue.

Tissue	UP–UP	UP–DOWN	DOWN–UP
adipose tissue	32,853	241	136
adrenal gland	143,475	71	0
animal ovary	138,094	1,853	0
appendix	139,817	378	0
bladder	35,560	135	337
bone marrow	262,579	107,999	15,561
cerebral cortex	759,042	14,773	454
colon	37,852	216	0
duodenum	134,912	450	0
endometrium	82,290	257	0
esophagus	45,240	838	503
fallopian tube	202,070	145	0
gall bladder	47,022	83	0
heart	29,213	13,131	1,441
kidney	144,093	927	240
liver	39,508	78,609	11,310
lung	80,042	0	0
lymph node	95,754	1,253	75
pancreas	11,300	106,338	11,105
placenta	142,998	1,789	759
prostate	142,588	0	0
rectum	65,735	1,598	0
salivary gland	38,962	16,418	519
skeletal muscle	43,104	107,190	15,291
skin	389,847	3,062	950
small intestine	93,035	292	0
smooth muscle	21,828	433	781
spleen	254,654	735	0
stomach	61,782	3	0
testis	4,079,755	33,648	0
thyroid	105,986	418	0
tonsil	119,525	1,866	0

Table S2: Tissue-specific human lncRNA–RNA interactions in LncRRIsearch. LncRNA and protein-coding genes with expression levels $\geq$ 1 FPKM in at least one tissue were used for the prediction. Expression levels were derived from RNA-seq data of the GTEx Consortium (Expression Atlas ID: E-MTAB-2919). Tissue-specific expression of lncRNA and protein-coding genes was detected by ROKU. UP–UP: Query and target RNAs are specifically up-regulated in the same tissue. UP–DOWN: Query RNAs are specifically up-regulated and target RNAs are down-regulated in the same tissue. DOWN–UP: Query RNAs are specifically down-regulated and target RNAs are up-regulated in the same tissue.

Tissue	UP–UP	UP–DOWN	DOWN–UP
adipose tissue	9,815	113	0
adrenal gland	58,999	1,710	1,293
artery	19,776	4,785	5,283
bladder	27,972	208	458
brain	1,836,197	168,590	28,347
breast	12,611	82	0
cervix	14,605	207	0
colon	76,519	160	0
esophagus	43,351	1,881	2,781
fallopian tube	48,427	264	28
heart	19,114	43,696	39,689
kidney	94,884	14,424	4,874
liver	73,506	86,114	19,653
lung	88,508	560	242
minor salivary gland	72,983	194	0
muscle	27,265	47,260	59,682
nerve	94,563	0	0
ovary	65,228	1,550	433
pancreas	36,928	43,435	20,091
pituitary gland	1,018,783	17,975	16,357
prostate	72,593	23	0
skin	72,596	938	0
small intestine	113,783	1,065	2,978
spleen	370,563	6,064	318
stomach	24,325	465	911
testis	6,074,963	141,685	29,144
thyroid	96,312	4	0
uterus	16,437	94	0
vagina	31,410	175	0
whole blood	122,180	292,259	80,697

Table S3: Tissue-specific human lncRNA–RNA interactions in LncRRIsearch. LncRNA and protein-coding genes with expression levels ≥ 1 FPKM in at least one tissue were used for the prediction. Expression levels were derived from RNA-seq data of the Illumina Body Map project (Expression Atlas ID: E-MTAB-513). Tissue-specific expression of lncRNA and protein-coding genes was detected by ROKU. UP–UP: Query and target RNAs are specifically up-regulated in the same tissue. UP–DOWN: Query RNAs are specifically up-regulated and target RNAs are down-regulated in the same tissue. DOWN–UP: Query RNAs are specifically down-regulated and target RNAs are up-regulated in the same tissue.

Tissue	UP–UP	UP–DOWN	DOWN–UP
adipose tissue	15,439	1,156	238
adrenal gland	1,016,541	4,823	0
brain	765,391	89,582	28,110
breast	60,423	1,149	1,046
colon	22,191	3,484	1,452
heart	31,064	30,966	5,617
kidney	108,372	4,352	99
leukocyte	138,245	64,896	29,478
liver	35,197	96,820	21,339
lung	112,074	13,014	198
lymph node	265,185	2,139	763
ovary	187,453	1,233	872
prostate	88,961	245	0
skeletal muscle	22,474	33,173	27,861
testis	1,496,550	2,339	468
thyroid	174,021	1,176	0

Table S4: Tissue-specific human lncRNA–RNA interactions in LncRRIsearch. LncRNA and protein-coding genes with expression levels ≥ 1 FPKM in at least one tissue were used for the prediction. Expression levels were derived from RNA-seq data of the NIH Epigenomics Roadmap project (Expression Atlas ID: E-MTAB-3871). Tissue-specific expression of lncRNA and protein-coding genes was detected by ROKU. UP–UP: Query and target RNAs are specifically up-regulated in the same tissue. UP–DOWN: Query RNAs are specifically up-regulated and target RNAs are down-regulated in the same tissue. DOWN–UP: Query RNAs are specifically down-regulated and target RNAs are up-regulated in the same tissue.

Tissue	UP–UP	UP–DOWN	DOWN–UP
adrenal gland	59,676	40,222	17,535
arm muscle	93,863	29,035	24,551
heart	43,675	21,952	35,004
kidney	4,961	0	0
large intestine	246,573	6,335	1,506
left kidney	16,743	322	0
left renal cortex	13	0	0
left renal pelvis	1	0	0
leg muscle	72,826	22,906	2,655
placenta	195,428	270,720	95,130
renal cortex	27	1	0
renal pelvis	1,268	5	0
right renal cortex	4	11	15
right renal pelvis	5	10	9
small intestine	165,452	9,418	5,304
spinal cord	444,145	63,530	18,784
stomach	797,010	397,214	48,404
thymus	197,179	149,015	62,082
trunk muscle	87,961	45,124	2,986

Table S5: Tissue-specific human lncRNA-RNA interactions in LncRRIsearch. LncRNA and protein-coding genes with expression levels $\geq$ 1 FPKM in at least one tissue were used for the prediction. Expression levels were derived from RNA-seq data of the FANTOM5 (Expression Atlas ID: E-MTAB-3358). Tissue- specific expression of lncRNA and protein-coding genes was detected by ROKU [1]. UP-UP: Query and target RNAs are specifically up-regulated in the same tissue. UP-DOWN: Query RNAs are specifically up-regulated and target RNAs are down-regulated in the same tissue. DOWN-UP: Query RNAs are specifically down-regulated and target RNAs are up-regulated in the same tissue.

Tissue	UP-UP	UP-DOWN	DOWN-UP
amygdala	15,795	0	0
artery	13,504	3,338	778
bone marrow	25,662	1,256	0
brain	27,938	2,134	0
brain meninx	26,805	339	0
breast	10,157	1,578	0
caudate nucleus	28,904	92	0
cerebellum	31,276	1,128	0
colon	40,409	4,332	733
diencephalon	14,357	127	0
dorsal thalamus	14,640	0	0
dura mater	28,598	509	0
epididymis	172,524	0	0
gall bladder	53,168	8	0
globus pallidus	13,751	0	0
heart	17,759	587	0
heart left ventricle	5,866	3,380	3,857
hippocampal formation	29,266	0	0
kidney	59,231	230	0
left cardiac atrium	13,131	2,573	0
locus ceruleus	41,395	0	0
lung	46,826	127	0
lymph node	22,527	1,760	179
medulla oblongata	30,630	0	0
middle frontal gyrus	24,975	0	0
middle temporal gyrus	51,127	0	0
mitral valve	17,455	259	0
occipital cortex	21,146	0	0
occipital lobe	28,109	392	0
olfactory apparatus	37,173	0	0
ovary	30,024	88	0
pancreas	13,389	1,802	0
parietal lobe	23,942	0	0
parotid gland	12,775	2,481	1,614
penis	38,738	115	0
pineal body	39,981	1,477	0
pituitary gland	51,384	1,312	0
placenta	68,779	2,869	2,402
prostate gland	51,944	0	0
pulmonary valve	21,124	0	0
putamen	23,468	39	0
seminal vesicle	29,451	319	0
smooth muscle tissue	12,178	0	0
spinal cord	20,759	0	0
spleen	31,310	433	0
submandibular gland	25,463	135	0
substantia nigra	872,391	117,275	20,893
testis	436,108	1,891	3,362
tongue	49,504	99	0

tricuspid valve	8,318	2,613	2,458
uterine cervix	57,786	0	0
uterus	39,140	329	0
vagina	8,263	1,972	870
vas deferens	32,713	83	0
vermiform appendix	57,838	52	0
zone of skin	14,298	30,884	39,864

Table S6: Tissue-specific mouse (C57BL/6 strain) lncRNA–RNA interactions in LncRRIsearch. LncRNA and protein-coding genes with expression levels $\geq$ 1 FPKM in at least one tissue were used for the prediction. Expression levels were derived from RNA-seq data (Expression Atlas ID: E-GEOD-74747). Tissue-specific expression of lncRNA and protein-coding genes was detected by ROKU. UP–UP: Query and target RNAs are specifically up-regulated in the same tissue. UP–DOWN: Query RNAs are specifically up-regulated and target RNAs are down-regulated in the same tissue. DOWN–UP: Query RNAs are specifically down-regulated and target RNAs are up-regulated in the same tissue.

Tissue	UP UP	UP DOWN	DOWN UP
brain	882,616	33,667	4,365
heart	34,261	12,343	1,903
kidney	51,147	18,553	2,526
liver	10,308	12,338	7,173
lung	78,734	960	0
spleen	97,199	1,647	0
testis	1,067,559	237,277	15,227
thymus	106,648	2,444	0
zone of skin	177,298	7,873	280

Table S7: Tissue-specific mouse (C57BL/6 strain) lncRNA–RNA interactions in LncRRIsearch. LncRNA and protein-coding genes with expression levels $\geq$ 1 FPKM in at least one tissue were used for the prediction. Expression levels were derived from RNA-seq data (Expression Atlas ID: E-MTAB-2801). Tissue-specific expression of lncRNA and protein-coding genes was detected by ROKU. UP–UP: Query and target RNAs are specifically up-regulated in the same tissue. UP–DOWN: Query RNAs are specifically up-regulated and target RNAs are down-regulated in the same tissue. DOWN–UP: Query RNAs are specifically down-regulated and target RNAs are up-regulated in the same tissue.

Tissue	UP UP	UP DOWN	DOWN UP
brain	809,114	9,121	10,690
colon	29,982	8,843	19,675
kidney	33,391	4,385	5,734
liver	30,579	7,313	9,805
lung	301,345	7,174	9,718
skeletal muscle tissue	19,392	28,766	34,535
spleen	540,540	4,402	3,317
testis	1,287,548	63,787	121,363

Table S8: Tissue-specific mouse (CD1 strain) lncRNA–RNA interactions in LncRRIsearch. LncRNA and protein-coding genes with expression levels $\geq$ 1 FPKM in at least one tissue were used for the prediction. Expression levels were derived from RNA-seq data (Expression Atlas ID: E-MTAB-2801). Tissue-specific expression of lncRNA and protein-coding genes was detected by ROKU. UP–UP: Query and target RNAs are specifically up-regulated in the same tissue. UP–DOWN: Query RNAs are specifically up-regulated and target RNAs are down-regulated in the same tissue. DOWN–UP: Query RNAs are specifically down-regulated and target RNAs are up-regulated in the same tissue.

Tissue	UP UP	UP DOWN	DOWN UP
brain	637,061	6,131	851
colon	33,410	3,517	994
heart	44,143	12,989	3,612
kidney	59,930	21,127	3,988
liver	200,175	58,651	1,496,387
lung	68,217	3,072	62
skeletal muscle tissue	224,246	65,916	1,515,522
spleen	82,844	1,368	0
testis	801,881	20,978	765

Table S9: Tissue-specific mouse (DBA/2J strain) lncRNA–RNA interactions in LncRRIsearch. LncRNA and protein-coding genes with expression levels $\geq$ 1 FPKM in at least one tissue were used for the prediction. Expression levels were derived from RNA-seq data (Expression Atlas ID: E-MTAB-2801). Tissue-specific expression of lncRNA and protein-coding genes was detected by ROKU. UP–UP: Query and target RNAs are specifically up-regulated in the same tissue. UP–DOWN: Query RNAs are specifically up-regulated and target RNAs are down-regulated in the same tissue. DOWN–UP: Query RNAs are specifically down-regulated and target RNAs are up-regulated in the same tissue.

Tissue	UP UP	UP DOWN	DOWN UP
brain	454,212	8,553	9,528
colon	28,682	3,847	7,700
heart	107,528	23,248	31,775
kidney	49,103	2,761	3,700
liver	15,428	6,021	10,561
lung	973,381	8,863	7,310
skeletal muscle tissue	7,331	29,919	55,671
spleen	263,663	3,921	5,975
testis	1,297,097	42,614	91,806

Table S10: Cytoplasmically localized RNA-RNA interactions in LncRRIsearch. The subcellular localization data derived from 15 human cell lines was imported from LncAtlas database. Cytoplasmic/nuclear relative concentration index (CN-RCI) is an index for cytoplasmic enrichment of the transcript over the nucleus in each cell line.

Cell line	CN-RCI ≥ 0	CN-RCI ≥ 1	CN-RCI ≥ 2	CN-RCI ≥ 3	CN-RCI ≥ 4
A549	1,530,837	227,195	7,618	62	10
GM12878	1,262,277	134,195	2,830	6	0
H1.hESC	2,469,846	283,630	15,508	949	50
HT1080	1,535,396	257,936	7,693	7	0
HUVEC	951,654	214,194	8,632	0	0
HeLa.S3	360,150	81,070	2,976	36	0
HepG2	801,561	155,568	7,868	19	0
IMR.90	183,027	59,572	3,530	27	0
K562	557,997	109,080	4,948	68	0
MCF.7	1,158,983	344,580	56,057	1,386	6
NCI.H460	443,793	56,273	5,012	1,643	714
NHEK	662,041	97,216	5,572	6	0
SK.MEL.5	583,537	108,987	21,204	903	12
SK.N.DZ	1,340,906	170,740	2,545	2	0
SK.N.SH	934,776	215,752	9,850	446	0

Table S11: Nuclear localized RNA-RNA interactions in LncRRIsearch. The subcellular localization data derived from 15 human cell lines was imported from LncAtlas database. Nuclear/cytoplasmic relative concentration index (NC-RCI) is an index for nuclear enrichment of the transcript over the cytoplasm in each cell line.

Cell line	NC-RCI ≥ 0	NC-RCI ≥ 1	NC-RCI ≥ 2	NC-RCI ≥ 3	NC-RCI ≥ 4
A549	2,854,143	857,660	169,834	18,821	608
GM12878	4,121,047	1,505,384	344,235	37,901	4,765
H1.hESC	5,388,914	1,286,287	189,727	8,203	249
HT1080	2,379,350	691,758	160,367	27,854	6,531
HUVEC	3,991,897	1,730,191	546,378	115,229	36,362
HeLa.S3	2,096,246	1,003,710	379,464	98,221	25,473
HepG2	3,904,403	1,954,918	669,198	203,484	52,860
IMR.90	626,709	281,056	111,556	33,496	11,750
K562	1,947,260	627,296	183,570	24,516	1,840
MCF.7	6,475,525	3,279,506	1,161,026	369,704	109,788
NCI.H460	2,967,624	1,125,249	237,991	57,194	10,474
NHEK	2,715,405	1,041,356	279,219	71,599	25,293
SK.MEL.5	2,516,688	1,193,915	469,466	133,562	38,060
SK.N.DZ	1,885,921	481,610	113,938	15,445	1,557
SK.N.SH	5,161,545	2,576,704	1,073,036	290,932	92,996

Table S12: Subcellular localized RNA-RNA interactions in LncRRIsearch. Sub-compartment level localization data derived from K562 human cell line was imported from LncAtlas database. In this dataset, five types of sub-compartment level RCI data (Chromatin/Nucleus, Nucleolus/Nucleus, Nucleoplasm/Nucleus, Cell membrane/Cytoplasm and Insoluble fraction/Cytoplasm) are provided.

Sub-compartment	RCI ≥ 0	RCI ≥ 1	RCI ≥ 2	RCI ≥ 3	RCI ≥ 4
insoluble	2,169,089	572,879	112,458	11,392	195
membrane	2,729,141	103,466	333	0	0
chromatin	2,050,713	734,717	140,832	22,048	4,783
nucleolus	2,648,951	660,302	108,818	10,656	789
nucleoplasm	3,764,804	1,006,744	152,825	32,226	4,725
